# Small RNA CjNC110 regulates the activated methyl cycle to enable optimal chicken colonization by *Campylobacter jejuni*

**DOI:** 10.1128/msphere.00832-24

**Published:** 2025-01-08

**Authors:** Brandon Ruddell, Alan Hassall, Orhan Sahin, Paul J. Plummer, Qijing Zhang, Amanda J. Kreuder

**Affiliations:** 1 Department of Veterinary Microbiology and Preventive Medicine, College of Veterinary Medicine, Iowa State University 1177 , Ames, Iowa, USA; 2 National Institute of Antimicrobial Resistance Research and Education (NIAMRRE), Iowa State University Research Park 658591 , Ames, Iowa, USA; 3 Department of Veterinary Diagnostic and Production Animal Medicine, College of Veterinary Medicine, Iowa State University 1177 , Ames, Iowa, USA; University of Nebraska Medical Center College of Medicine, Omaha, Nebraska, USA

**Keywords:** *Campylobacter*, activated methyl cycle, L-methionine, small RNAs, chicken colonization

## Abstract

Post-transcriptional gene regulation by non-coding small RNAs (sRNAs) is critical for colonization and survival of enteric pathogens, including the zoonotic pathogen *Campylobacter jejuni*. In this study, we utilized *C. jejuni* IA3902 (a representative isolate of the sheep abortion clone) and *C. jejuni* W7 (a highly motile variant of NCTC 11168, a human gastroenteritis strain) to further investigate regulation by sRNA CjNC110. Both motility and autoagglutination ability were confirmed to be phenotypes of conserved regulation by CjNC110. However, we demonstrated that W7∆CjNC110 does not change chicken colonization levels compared to W7 wild type, directly contrasting IA3902∆CjNC110, which had decreased colonization ability. Subsequently, we determined strain-specific phenotype variation between W7∆CjNC110 and IA3902∆CjNC110 when examining intracellular L-methionine (L-met) levels controlled by the activated methyl cycle (AMC). We hypothesized that the presence of a secondary system for L-met production conferred by MetAB in W7 but not IA3902 might explain the difference in both chicken colonization and L-met availability. Insertion of *metAB* within IA3902∆CjNC110 (naturally absent) restored intracellular L-met levels in IA3902∆CjNC110::metAB and overcame the colonization defect that resulted from mutagenesis of CjNC110 in IA3902. Deletion of metAB in W7∆CjNC110 (naturally present) led to a decrease in L-met in W7∆CjNC110∆metAB and a colonization defect which was otherwise masked in W7∆CjNC110. Our results indicate that regulation of the AMC leading to altered L-met availability is a conserved regulatory function of CjNC110 in *C. jejuni* and confirm that L-met generation via the AMC as activated by CjNC110 is critical for optimal host colonization.

**IMPORTANCE:**

During this study, the regulatory action and conservation of function of CjNC110 between two different zoonotically important *Campylobacter jejuni* strains were examined. Critically, this work for the first time reveals regulation of L-methionine (L-met) production within the activated methyl cycle (AMC) by small RNA (sRNA) CjNC110 as a key factor driving *C. jejuni* optimal chicken colonization. As a growing body of evidence suggests that maintenance of L-met homeostasis appears to be critical for *C. jejuni* colonization, interventions targeting the AMC could provide a critical control point for therapeutic drug options to combat this zoonotic pathogen. Our results also indicate that even for conserved sRNAs such as CjNC110, strain-specific differences in phenotypes regulated by sRNAs may exist, independent of conserved regulatory action. Depending on the strain examined and accessory genomic content present, conserved regulatory actions might be masked, thus investigation in multiple strains may be warranted.

## INTRODUCTION

*Campylobacter jejuni* is one of the primary agents of human gastroenteritis, and foodborne transmission is often associated with the consumption of contaminated chicken or raw milk (1–4). Within *C. jejuni*, whole-genome sequencing studies have demonstrated high synteny between genomes of various strains (5–7), but subtle differences, such as gene gain or loss, single nucleotide polymorphisms (SNPs), and hypervariable regions, frequently occur in *C. jejuni* genomes, resulting in phenotypic differences between strains (7–10). For example, *C. jejuni* sheep abortion (SA) clone has emerged as one of the most common pathogens causing ovine abortions in the United States and is also associated with human gastroenteritis outbreaks due to transmission by the foodborne route (1, 11, 12). A multi-omics study comparing *C. jejuni* IA3902 (representative of clone SA) to the genetically syntenic but non-abortifacient strain *C. jejuni* NCTC 11168 (originated from human gastroenteritis) revealed numerous unique features of IA3902 despite minimal differences in genetic content between the two strains (7). In particular, IA3902 was noted to have differential expression compared to NCTC 11168 of conserved mRNAs and proteins connected to iron uptake systems, cell motility, flagellar modification, and amino acid utilization, suggesting unique adaptations for host specificity, colonization, and translocation between strains (7). Phenotypically, IA3902 is highly invasive and induces systemic infection, while NCTC 11168 is primarily a gut colonizer (13, 14). Taken together, these observations suggest that subtle differences in genetic contents and/or regulation of gene expression may play a key role in differentiating virulence properties between *C. jejuni* strains.

Gene regulation in *C. jejuni* remains understudied compared to the model organisms *Escherichia coli* and *Salmonella*; however, recent studies have demonstrated that *C. jejuni* expresses numerous non-coding small RNAs (sRNAs) that could help explain observed differences in gene expression and phenotypes between strains (15–19). sRNAs act post-transcriptionally by binding to target mRNAs, most typically within the 5′-untranslated region (UTR), resulting in changes to the stability of the mRNA transcript and thereby activating or repressing protein translation (20–22). A hallmark study by Dugar et al. revealed the sRNA transcriptional profiles of four *C. jejuni* strains using RNAseq and northern blotting, establishing the presence of both a core conserved sRNA repertoire and strain-specific sRNAs (15). *C. jejuni* strains have also been demonstrated to differentially express sRNAs in the sheep gallbladder and chicken ceca, indicating that *in vivo* post-transcriptional modulation by sRNAs is critical (17, 18).

In *C. jejuni*, recent studies by us have demonstrated that mutagenesis of conserved sRNA CjNC110 in strain IA3902 resulted in changes to numerous virulence-associated phenotypes, including intracellular L-methionine (L-met) availability, motility, autoagglutination, hydrogen peroxide (H_2_O_2_) sensitivity, and chicken colonization ability (23, 24). However, based on the documented genotypic and phenotypic differences between IA3902 and NCTC 11168 (7, 14, 25), it is unclear if all these phenotypic changes are conserved across strains, especially in relation to L-met availability and chicken colonization ability. Specifically, we have previously demonstrated significant strain-specific differences in the ability to colonize chickens and produce L-met when *luxS*, a key enzyme of the activated methyl cycle (AMC), is deleted. The core structure of the AMC, including LuxS, is conserved in both IA3902 and NCTC 11168 and is responsible for the generation of L-met, S-adenosylmethionine (SAM), and the toxic intermediate S-adenosylhomocysteine (SAH) (26–29). LuxS plays a key role in the detoxification of SAH and, in *C. jejuni*, has been connected to virulence traits important for fitness and/or colonization ability (30). In NCTC 11168, the deletion of *luxS* leads to no alteration in chicken colonization ability despite disruption of the core AMC; in IA3902, the deletion of *luxS* leads to near-complete abolishment of the ability to colonize chickens (31). We demonstrated that these differences are associated with the presence of a secondary system conferred by MetAB for generation of key precursors directly leading to increased L-met in NCTC 11168 when the AMC is disrupted; this ability is lost in strains such as IA3902 which naturally lacks the MetAB system (31).

As sRNA CjNC110 has been shown to regulate the phenotypic expression of intracellular L-met levels and chicken colonization, we hypothesized that differences may exist in these phenotypes (and potentially others as well) between strains IA3902 and NCTC 11168 when CjNC110 is deleted. Despite clearly demonstrating phenotypic differences between strains with the disruption of CjNC110, the work presented herein ultimately serves to directly connect sRNA CjNC110 as a conserved regulator of the AMC and further supports AMC metabolic turnover as critical for *C. jejuni* colonization. This study is unique in demonstrating the positive regulation of the AMC by sRNA CjNC110 and conserved targeting of flagella-associated gene products by CjNC110, which are also important for *C. jejuni* pathogenesis.

## RESULTS

### Deletion of CjNC110 in W7, a highly motile variant of NCTC 11168, does not affect growth rate in culture media

Following the deletion of CjNC110 in a highly motile variant of NCTC 11168 (hereafter referred to as W7) through homologous recombination from previously developed constructs (23), growth curves were conducted using three independent experiments with wildtype (WT), respective isogenic mutants (W7∆CjNC110, W7∆luxS, and W7∆luxS∆CjNC110) and complement (W7∆CjNC110c) as previously described (23). As CjNC110 is located immediately downstream of *luxS*, W7∆luxS and W7∆luxS∆CjNC110 were utilized as previously described to ensure a lack of polar effects after deleting CjNC110 (23). Growth in Mueller-Hinton (MH) broth over 30 h was analyzed by collecting average optical density readings (*A*_600_) and colony-forming units (CFU) per milliliter counts (Fig. S1). For CFU per milliliter, two-way analysis of variance (ANOVA) demonstrated no statistical (*P* > 0.05) difference over time between W7 strains. However, at 28 and 30 h, *A*_600_ readings significantly (*P* < 0.05) decreased for W7∆CjNC110, while complementation (*W7*∆CjNC110c) corrected this phenotypic defect; both trends were similarly noted for IA3902 CjNC110 isogenic mutants previously (23).

After determining that W7 WT and W7 isogenic mutants grew at similar rates, our next step was to validate sRNA CjNC110 expression *in vitro*. CjNC110 has previously been detected by northern blotting in multiple strains of *C. jejuni*, including NCTC 11168 (15, 23). During this study, northern blot analysis demonstrated that CjNC110 is expressed as two separate bands in W7 WT, absent in W7ΔCjNC110, and re-expressed in W7ΔCjNC110c, matching previously reported results for *C. jejuni* IA3902 WT and W7 WT (Fig. S2) (15, 23).

### Deletion of CjNC110 in W7 does not affect chicken colonization ability, directly contrasting the decreased chicken colonization seen with deletion in IA3902

**Fig 1:**
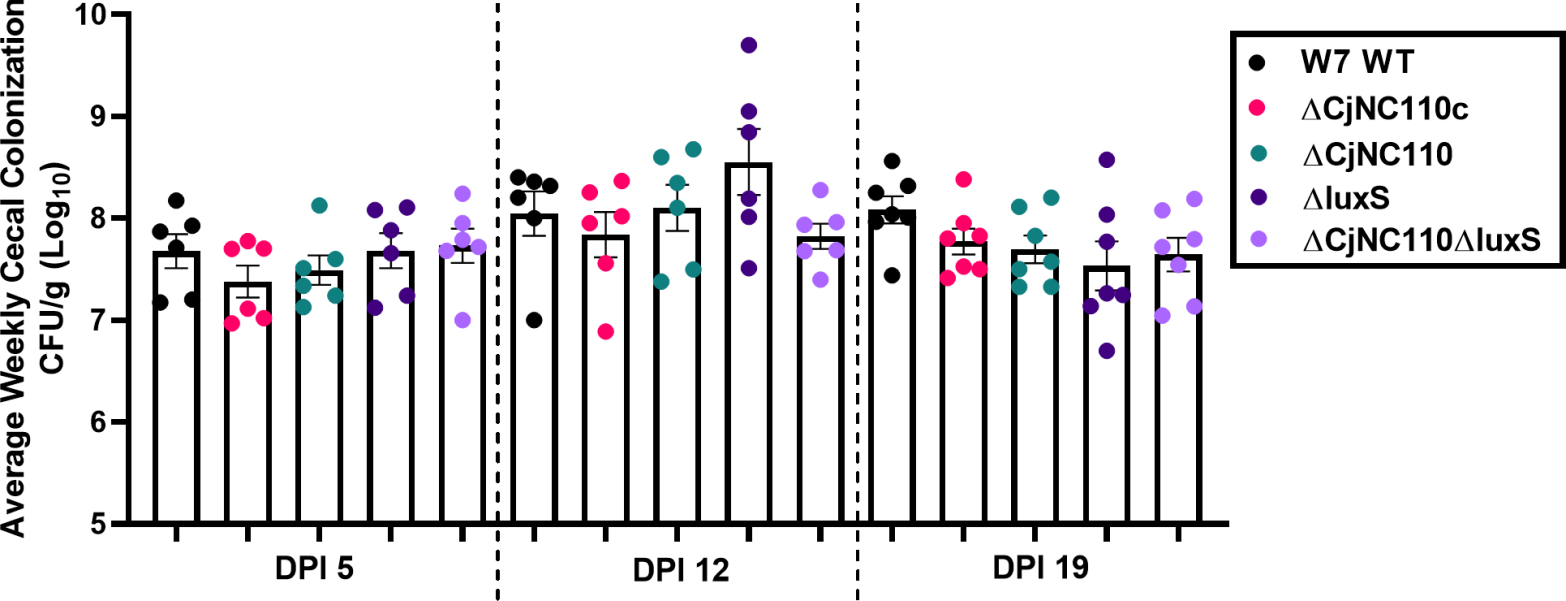
W7 CjNC110 and *luxS* background mutants colonize at comparable levels to W7 WT at DPI 5, 12, and 19 (mean ± SEM). The ability of W7∆CjNC110 to colonize at comparable levels to WT directly contrasts IA3902∆CjNC110, which had hindered colonization at each DPI previously (23). Each dot represents an individual bird, and each color indicates the strain utilized displayed on the right (black box). Each bar represents the average CFU per g (log_10_), with a minimum of six birds per strain each week. One-way ANOVA demonstrated no statistical difference in colonization at each DPI (*P* > 0.05).

Based on the decreased colonization ability of IA3902∆CjNC110 noted in previous studies (23), we elected to first test chicken colonization using W7 WT and isogenic mutants W7∆CjNC110, W7∆*CjNC110c*, W7∆*luxS*, and W7∆luxS∆CjNC110. Following oral challenge with approximately 10^7^ CFU of each *C. jejuni* strain (Fig. S3), six chickens were euthanized at 5, 12, and 19 days post-inoculation (DPI), and cecal contents were collected for CFU per gram determination as previously described (23). One-way ANOVA demonstrated that all strains were able to colonize consistently at comparable levels to W7 WT with no statistical difference at any DPI (*P* > 0.05; Fig. 1). This finding directly contrasts the previous work performed in IA3902, as IA3902ΔCjNC110 had significantly decreased colonization ability at DPI 5, 12, and 19 compared with WT IA3902 (23). The W7ΔluxS chicken ceca colonization trends were consistent with previously reported results, as colonization was not altered when compared to WT (23). Inactivation of both *luxS* and *CjNC110* also did not result in alteration to colonization ability in W7∆luxS∆CjNC110.

### W7ΔCjNC110 bolsters motility and hinders autoagglutination, similar to IA3902ΔCjNC110, indicating a conserved role for CjNC110 regulation of flagella-associated phenotypes

**Fig 2:**
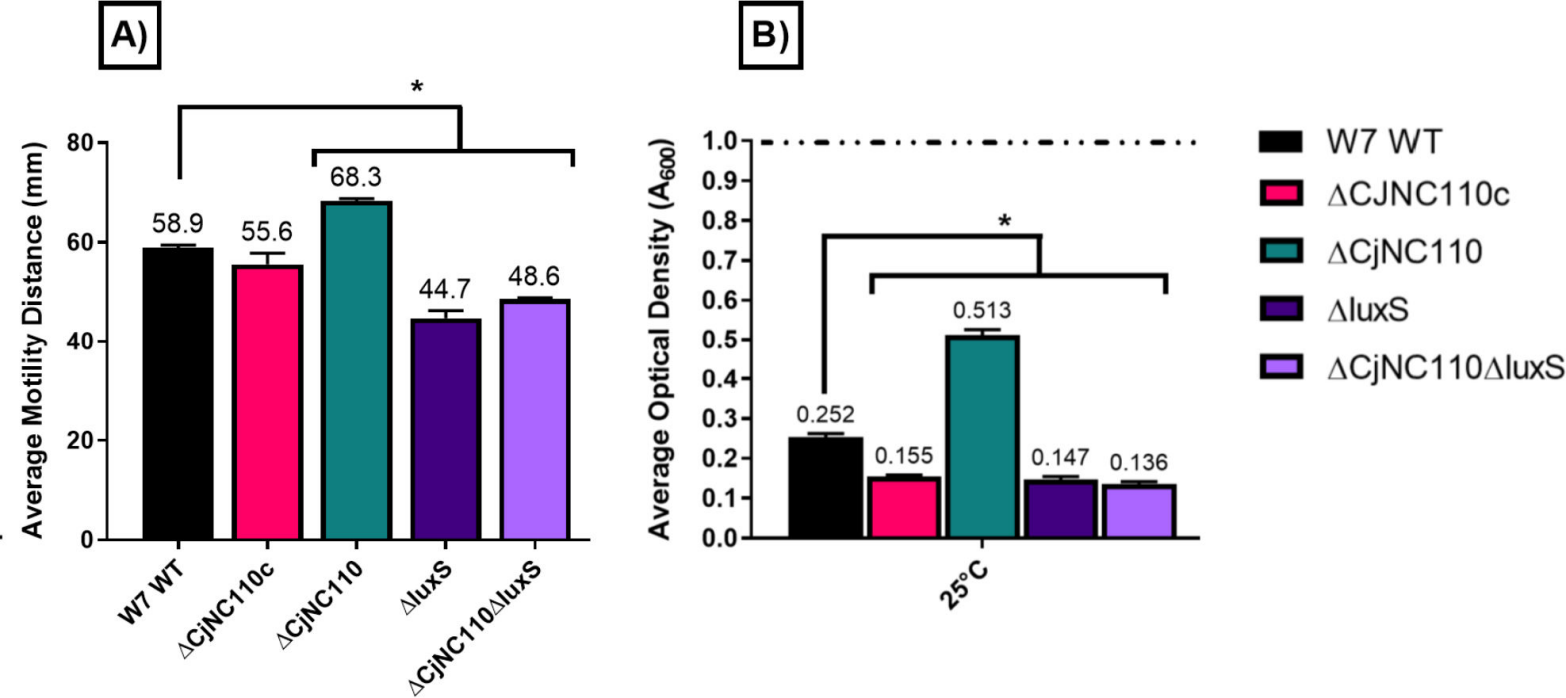
W7∆CjNC110 increases motility and decreases autoagglutination, matching the biological trends of IA3902 isogenic mutants (23) (mean ± SEM). Colored bars indicate the average (A) motility in millimeter or (B) autoagglutination determined by *A*_600_ at 24 h, using a minimum of at least three technical replicates from three independent studies. An increase in *A*_600_ correlates to decreased autoagglutination ability, and a decrease in *A*_600_ correlates to increased autoagglutination ability. For statistical analysis, one-way or two-way ANOVA with Tukey’s multiple comparison test was performed for each assay when appropriate. Significance (*P* < 0.05) is denoted by “*” and black lines when comparing W7 WT to all other strains.

To further determine the regulatory basis for the observed difference in colonization ability between strains W7 and IA3902 with inactivation of CjNC110, we then set out to test known modulated phenotypes of CjNC110 that have been previously suggested to correlate with *C. jejuni* chicken colonization ability (32). First, motility and autoagglutination assays were performed as previously described (23). When compared to W7 WT, W7ΔCjNC110 motility significantly increased (*P* < 0.05), while W7ΔCjNC110c restored the motility phenotype to wild type (*P* > 0.05; Fig. 2A). These findings match motility phenotype trends previously observed after inactivation of CjNC110 in IA3902 (23). In W7ΔluxS and W7ΔCjNC110ΔluxS, motility significantly decreased (*P* < 0.05); this was also consistent with IA3902 *luxS* isogenic mutants as previously described (23) and indicates a lack of polar effects between mutation of CjNC110 and *luxS*. These results reveal that CjNC110 displays a conserved role in the repression of motility in *C. jejuni*.

Autoagglutination ability significantly (*P* < 0.05) differed and had similar biological trends when comparing W7 WT and W7 isogenic mutants at both 25 (Fig. 2B) and 37°C (Fig. S4). W7ΔCjNC110 autoagglutination levels significantly (*P* < 0.05) decreased when compared to W7 WT; W7ΔCjNC110c restored the autoagglutination defect and significantly (*P* < 0.05) increased autoagglutination ability compared to WT. W7ΔCjNC110 and W7ΔCjNC110c autoagglutination trends matched previously examined isogenic IA3902 mutants, although overcompensation was not observed previously in the IA3902 complement (23). In contrast, W7ΔluxS and W7ΔCjNC110ΔluxS both significantly (*P* < 0.05) increased in autoagglutination ability; this is consistent with the results reported for the IA3902 Δ*luxS* background strains (23). The increased autoagglutination phenotype of W7ΔluxS background mutants compared to decreased autoagglutination in W7ΔCjNC110 again indicated that polar effects did not occur. These results demonstrate a conserved role for CjNC110 in activation of autoagglutination in *C. jejuni* in addition to repression of motility, both of which are highly associated with modulation of flagella-associated gene products.

### W7ΔCjNC110 H_2_O_2_ sensitivity and intracellular L-met concentrations are comparable to W7 WT, contrasting significant differences previously seen in IA3902ΔCjNC110

**Fig 3:**
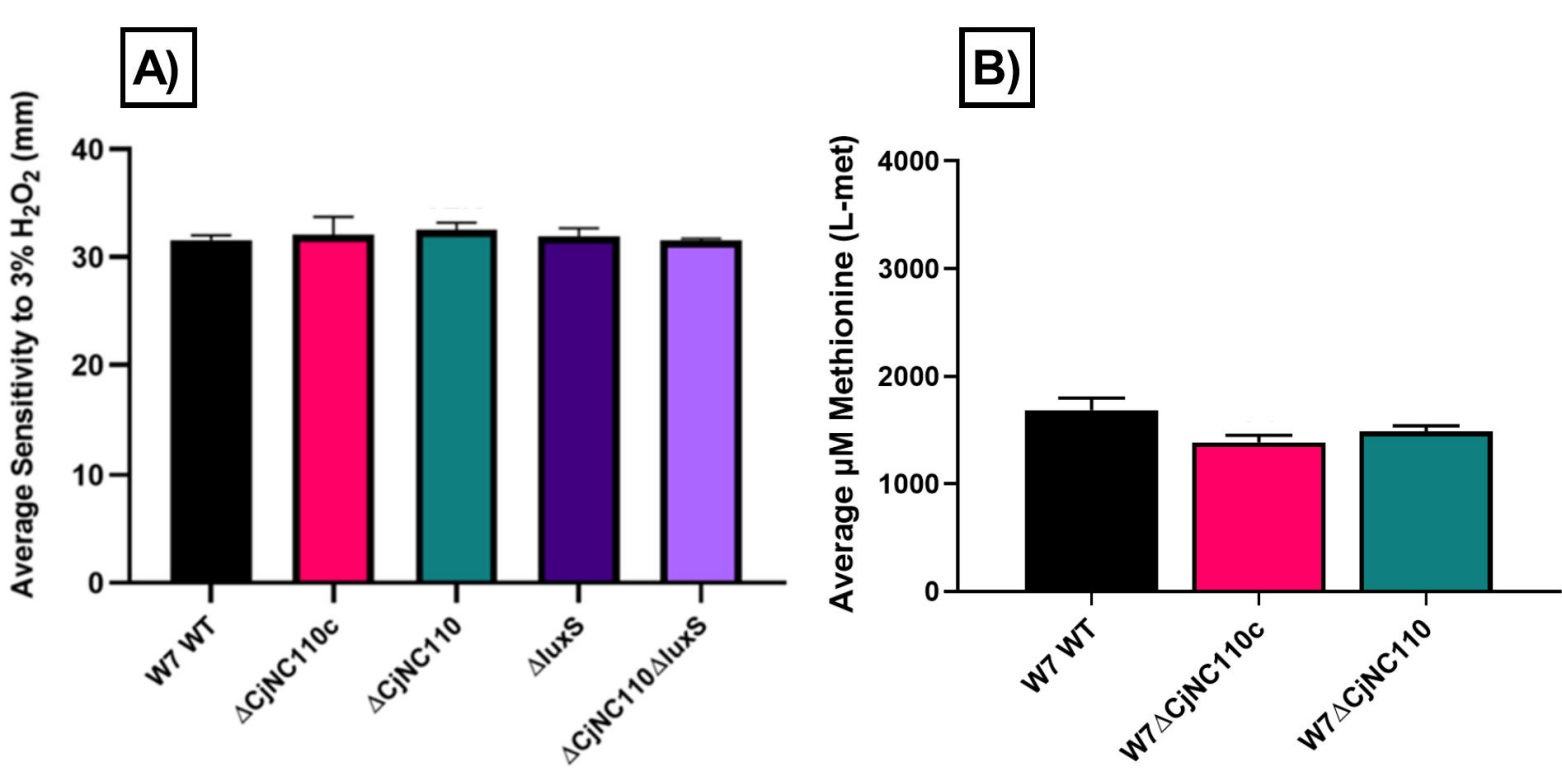
(A) W7ΔCjNC110 had comparable hydrogen peroxide (H_2_O_2_) sensitivity and (B) intracellular L-met levels relative to W7 WT, contrasting the increased sensitivity to H_2_O_2_ and decreased L-met production in IA3902ΔCjNC110 demonstrated previously (23). Strains utilized are indicated at the bottom (*x*-axis). Colored bars indicate the average sensitivity to (**A**) H_2_O_2_ or (**B**) L-met metabolite concentration of each strain tested using three technical replicates from three independent studies per assay (mean ± SEM). For statistical analysis, each assay was analyzed independently, and each strain was compared to the other. One-way ANOVA tests demonstrated no significant difference per assay (*P* > 0.05).

Next, a hydrogen peroxide (H_2_O_2_) sensitivity assay was performed as previously described (23). When comparing W7 WT and isogenic mutants, there was no significant difference noted in H_2_O_2_ sensitivity (*P* > 0.05) for any mutant tested (Fig. 3A). Previously, IA3902 CjNC110 background mutants demonstrated increased sensitivity to H_2_O_2_ when compared to IA3902 WT (23). A time-resolved fluorescence energy transfer (TR-FRET) assay was then performed as previously described to determine the intracellular concentration of L-met in *C. jejuni* (24, 31). The data presented in Fig. 3B demonstrate that W7∆CjNC110 and W7∆CjNC110c had no significant change (*P* > 0.05) in L-met levels compared to WT. Previously, IA3902∆CjNC110 demonstrated decreased L-met production (24), directly contrasting the results reported here for W7∆CjNC110. W7∆luxS was not tested in this assay as it was previously shown to exhibit WT L-met levels (31). Both the H_2_O_2_ stress response and L-met availability are considered critical for *C. jejuni* survival within the host environment (33, 34); therefore, further investigation of these key *in vitro* phenotypes was warranted to determine if either contributes to the observed differences in chicken colonization between IA3902∆CjNC110 and W7∆CjNC110 as described above.

### Computational analyses reveal both a predicted core CjNC110 regulon and potential contrasting CjNC110 mRNA interactions between IA3902 and W7

To generate insights into how CjNC110 regulates the H_2_O_2_ stress response and L-met concentration, computational analysis using IntaRNA, RNAup, and BlastN was performed to elucidate putative regulatory targets of CjNC110 (35, 36). We hypothesized that the phenotypic differences seen between the two strains could be explained either by changes in the accessory genes present or via SNPs that might alter the outcome of regulation by CjNC110. The global IntaRNA analysis revealed a core top regulatory network with 155 5′UTR and 170 3′UTR (total of 325) predicted regulatory CjNC110-mRNA interactions shared by *C. jejuni* W7 and IA3902. In both W7 and IA3902, motility (e.g., *cetB*, *flaG*, *fliN*, *flgK*, *fliI*, *flgS*, *and cheQ*) and flagellar-glycosylation-associated mRNAs (e.g., *pglABCEJK*, *ptmA*, and *cgpA*) were computationally predicted via the global search to be regulated by CjNC110 (File S1; Table 1). Additionally, global IntaRNA analysis revealed conserved H_2_O_2_ stress response and AMC mRNAs targeted by CjNC110 (File S1; Table 1). Based upon the global IntaRNA search discoveries and the observed differences in phenotypes reported above, specific putative mRNA targets related to the H_2_O_2_ stress response and the AMC were then further analyzed using both IntaRNA and RNAup single target input mode (35, 36).

**TABLE 1:** CjNC110 regulatory mRNA interactions are predicted within the H_2_O_2_ stress response in both *C. jejuni* IA3902 and W7

Gene	Gene ID IA3902	Gene ID 11168	UTR	mRNA nt^*a*^	sRNA nt^*b*^	Binding energy (kcal/mol)^*c*^	Strain	Program^*d*^
*sodB*	CJSA_0159	Cj0169	5'	−66 to −50	**13–29**	−7.59	Both	IntaRNA
*perR*	CJSA_0296	Cj0322	5'	−57 to −48	**13–22**	−6.34	Both	RNAup
*ahpC*	CJSA_0308	–^*e*^	3'	32–34	**19–21**	−3.47	IA3902	IntaRNA
*ahpC*	–	Cj0344	3'	6–14	125–133	−2.85	W7	IntaRNA
*cosR*	CJSA_0329	Cj0355c	5'	−66 to −51	**44–58**	−3.31	Both	RNAup
*Cjfur*	CJSA_0373	Cj0400	5'	68–72	130–134	−1.89	Both	IntaRNA
*tpx*	CJSA_0735	Cj0779	5'	−51 to −47	**18–22**	−4.35	Both	RNAup
*katA*	CJSA_1319	Cj1385	5'	73–78	92–97	−3.66	Both	RNAup

^*a*^ Nucleotides (nt) ±75 nt from ribosomal-binding site.

^*b*^ Nucleotides from the beginning of sRNA; stem loop 1 (SL1) of CjNC110 is nt 15–22, and stem loop 2 (SL2) is nt 48–54; interactions with these SL regions are in bold.

^*c*^ Free energy required to unfold the interaction site for accessibility (deltaG or kcal/mol), a lower kcal/mol is more favorable; mRNAs with binding energy scores in the top 325 CjNC110 partner list determined by IntaRNA global search are underlined.

^*d*^ The lowest free energy score is reported using either IntaRNA (35) or RNAup (36).

^*e*^ “–” indicates either that no gene is present in that strain or that the predicted interactions differed enough between strains that each is listed on a separate line..

Single target computational analyses via RNAup and IntaRNA of the H_2_O_2_ stress response transcripts demonstrated that in both W7 and IA3902, CjNC110 was predicted to have identical binding energy scores and seed interactions with several of the central players of the oxidative stress response (37–39) (Table 1). Previously, RNAseq analysis demonstrated IA3902∆CjNC110 downregulated superoxide dismutase (*sodB*) and thiol peroxidase (*tpx*) mRNAs when compared to IA3902 WT (23). As *tpx* and *sodB* share 100% homology between strains and have identical binding site predictions, these results strengthen the hypothesis that CjNC110 may activate their expression, but it does not explain the different phenotypes between strains. The primary difference in computational results between W7 and IA3902 related to known oxidative stress genes was the predicted interactions between CjNC110 and *ahpC*, which resulted in different binding energy scores and binding locations. While AhpC has been demonstrated to play a role in H_2_O_2_ resistance in *E. coli*, when investigated in *C. jejuni* 81116, AhpC did not show antioxidant activity against H_2_O_2_ (40). Thus, it is unlikely that a potential difference in CjNC110 regulation of *ahpC* would be sufficient to explain the observed phenotypic differences in H_2_O_2_ sensitivity between *C. jejuni* strains.

Computational analyses also revealed multiple putative mRNA regulatory partners of CjNC110 connected to the AMC which is responsible for L-met production (28) and the secondary utilization of AMC by-products L-met and SAM (Table 2). Conserved binding sites were predicted at SL1 or SL2 of CjNC110 to the 5′-UTR regions of several key biosynthesis AMC mRNAs, including *pfs*, *luxS*, *metF*, and *metE* as well as secondary pathway genes *aspB* and *hom* used for aspartate conversion. Conserved binding sites and binding energies were also predicted for L-met transport mRNAs within the key MetNIQ transport system including *metN* and two genes predicted to be *metQ* methionine-binding protein homologs; *C. jejuni* harbors a total of four predicted homologs of MetQ with DNA/protein sequences that vary between each other (41, 42). Three copies of MetQ are located immediately adjacent to each other within a single operon upstream of MetNI (CJSA_0728-CJSA_0726), and a fourth MetQ homolog is present immediately adjacent to and within the same operon as MetE (CJSA_1138). Differential mRNA-binding sites and binding energies between W7 and IA3902 were identified for putative methyltransferase CJSA_0238 and one of the putative methionine-binding protein *metQ* homologs (CJSA_1138). While one or both may represent a potential explanation for the difference in phenotypes, a lack of previous research on these genes in *C. jejuni* precluded a clear hypothesis that could be readily tested.

**TABLE 2:** CjNC110 regulatory mRNA interactions are predicted within the AMC as well as secondary L-met generation and transport systems in both *C. jejuni* IA3902 and W7

		Gene^*a*^	Gene ID IA3902	Gene ID 11168	UTR	mRNA nt^*b*^	sRNA nt^*c*^	Binding energy (kcal/mol)^*d*^	Strain	Program^*e*^
AMC biosynthesis	Core	*pfs*	CJSA_0108	Cj0117	5′	6–12	**16–22**	−5.42	Both	IntaRNA
		*methyltransferase*	CJSA_0238	–^*g*^	5′	20–26	69– 75	−10.36	IA3902	IntaRNA
		*methyltransferase*	–	Cj0261c	5′	58–64	**48–54**	−5.28	W7	IntaRNA
		*metK*	CJSA_1038	Cj1096c	5′	−25 to −1	90–108	−4.01	Both	RNAup
		*luxS*	CJSA_1136	Cj1198	5′	−74 to −62	**11–23**	−7.10	Both	RNAup
		*metE* ^a1^	CJSA_1139	Cj1201	5′	19–23	**18–22**	−4.24	Both	RNAup
		*metF* ^a1^	CJSA_1140	Cj1202	5′	10–23	**10–23**	−6.78	Both	RNAup
	Secondary	*metAB* ^*f*^	–	Cj1729-30c	CDS^*f*^	1,606–1,612	**18–24**	−2.06	W7 only	RNAup
		*hom*	CJSA_0140	Cj0149c	5′	52–58	**17–23**	−6.42	Both	IntaRNA
		*aspB*	CJSA_0718	Cj0726c	5′	52–58	**48–54**	−4.96	Both	IntaRNA
		*heuR*	CJSA_1321	Cj1387c	5′	7–13	64–70	−3.01	Both	RNAup
		*metC*	CJSA_1324	Cj1392	5′	66–69	**18–21**	−4.35	Both	RNAup
Transport		*metQ3* ^a2^	CJSA_0726	Cj0770c	5′	−10 to −16	**14–21**	−2.28	Both	IntaRNA
		*metQ2* ^a2^	CJSA_0727	Cj0771c	5′	−36 to −20	23–40	−5.55	Both	RNAup
		*metQ1* ^a2^	CJSA_0728	Cj0772c	5′	67–74	**48–54**	−2.99	Both	RNAup
		*metI* ^a3^	CJSA_0729	Cj0773c	5′	−24 to −21	**19–22**	−1.91	Both	RNAup
		*metN* ^a3^	CJSA_0730	Cj0774c	5′	47–66	**4–25**	−5.24	Both	RNAup
		*metQ4* ^a1^	CJSA_1138	–	5′	35–41	**16–22**	−5.06	IA3902	IntaRNA
		*metQ4* ^a1^	–	Cj1200	5′	53–59	**16–22**	−5.04	W7	IntaRNA

^*a*^ a1/a2/a3, predicted operon(s).

^*b*^ Nucleotides (nt) ±75 nt from ribosomal-binding site.

^*c*^ Nucleotides from the beginning of sRNA; stem loop 1 (SL1) of CjNC110 is nt 15–22, and stem loop 2 (SL2) is nt 48–54; interactions with these SL regions are in bold.

^*d*^ Free energy required to unfold the interaction site for accessibility (deltaG or kcal/mol), a lower kcal/mol is more favorable; mRNAs and binding energy scores that were in the top 325 CjNC110 partner list determined by IntaRNA global search are underlined.

^*e*^ The lowest free energy score is reported using either IntaRNA (35) or RNAup (36).

^*f*^ The genes metAB are absent in *C. jejuni* IA3902; CDS, coding sequence of gene.

^*g*^ “–” iindicates either that no gene is present in that strain or that the predicted interactions differed enough between strains that each is listed on a separate line.

The most critical difference identified between strains related to the AMC was the presence of *metA* and *metB* in W7 but not IA3902; these enzymes provide downstream production of L-homocysteine via aspartate conversion that feeds into the AMC as an alternate secondary pathway for L-met production (28, 31). Given that we have previously demonstrated role for MetAB in maintaining chicken colonization ability following disruption of the AMC and a lack of otherwise identified strong candidate for differential mRNA interactions associated with H_2_O_2_ sensitivity and the AMC, we therefore elected to utilize the *metAB* system to test if CjNC110 is a critical regulator of the AMC (31). We hypothesized that CjNC110 directly or indirectly regulates the core conserved AMC in *C. jejuni,* but the secondary MetAB system present in W7 masks the loss of this positive regulatory action in W7∆CjNC110.

### The MetAB system rescues IA3902∆CjNC110 L-met production and chicken colonization ability, confirming CjNC110 as a key regulator of the AMC and *C. jejuni* colonization

**Fig 4:**
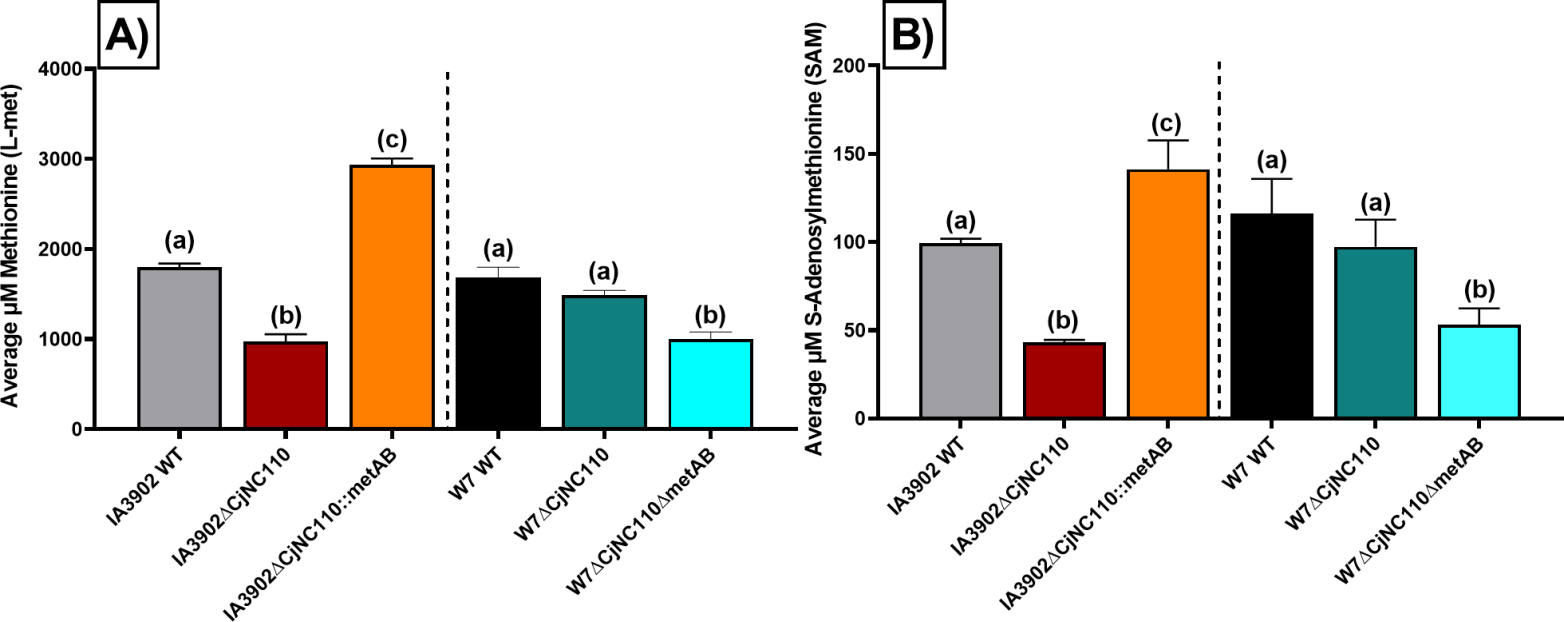
IA3902∆CjNC110::metAB restores L-met and SAM production in IA3902∆CjNC110, while W7∆CjNC110∆metAB reduces L-met and SAM concentration to comparable levels observed for IA3902∆CjNC110 *in vitro*. (**A**) Intracellular L-met and (B) SAM were extracted from cell cultures at the early stationary phase of growth (12 h). The strains utilized are indicated at the bottom (*x*-axis). Each bar represents the average intracellular metabolite concentration of each strain tested using three technical replicates from three independent studies (mean ± SEM). For statistical analysis, each strain was compared to all the other strains using one-way ANOVA with Tukey’s multiple comparison test for each metabolite. Overlap in letters above each bar indicates no significance (*P* >  0.05) detected.

To test our hypothesis, previously constructed *metAB* deletion and insertion mutants (31) were used to generate IA3902∆CjNC110::metAB (insertion) and W7∆CjNC110∆metAB (deletion). L-met and SAM concentrations in these constructs were then examined using TR-FRET assays as previously described (31). IA3902∆CjNC110::metAB significantly (*P* < 0.05) increased L-met production compared to WT strains and restored the L-met decreased production created by inactivation of CjNC110 in IA3902 (Fig. 4A). In contrast, W7∆CjNC110∆metAB significantly (*P* < 0.05) reduced L-met levels, matching the IA3902∆CjNC110 phenotype, as there was no significant (*P* > 0.05) difference in L-met production between W7∆CjNC110∆metAB and IA3902∆CjNC110. Similar biological trends also occurred for downstream SAM production as expected (Fig. 4B; Fig. S5). Thus, we successfully demonstrated that the addition of the MetAB system enables recovery of L-met and SAM levels in IA3902∆CjNC110, while the deletion of MetAB in W7∆CjNC110 depletes L-met and SAM levels *in vitro*, confirming a key role for CjNC110 in regulation of the core conserved AMC (present in both strains) which is masked in W7 by the presence of the secondary MetAB system (present only in W7).

**Fig 5:**
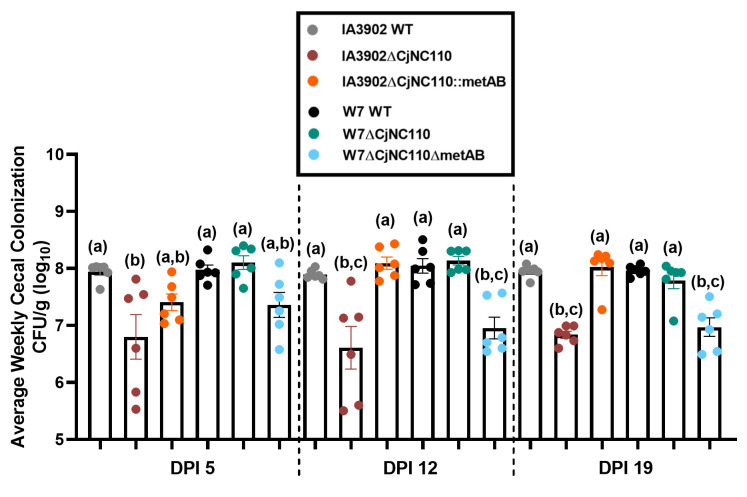
The reacquired MetAB system bolsters chicken colonization levels of IA3902ΔCjNC110 to comparable levels to W7ΔCjNC110 and IA3902 WT, and mutagenesis of *metAB* hinders W7ΔCjNC110 colonization at DPI 12 and 19. Each dot represents an individual bird, and each color indicates the strain utilized displayed at the top (black box). Each bar represents the average CFU per g (log_10_), with a minimum of six birds per strain each week (mean ± SEM). For statistical analysis, each strain was compared to all the other strains using one-way ANOVA with Tukey’s multiple comparison test at each DPI. Overlap in letters above each bar indicates no significance (*P* >  0.05) was detected.

The chicken colonization study was then repeated to test if the strain-specific differences in colonization between W7∆CjNC110 and IA3902∆CjNC110 could be explained via the observed *in vitro* phenotypic difference in L-met production. All strains were able to colonize but at different levels at DPI, 5, 12, and 19 (Fig. 5). As anticipated, IA3902∆CjNC110 again had significantly reduced colonization at DPI 5, 12, and 19 (*P* < 0.05). By DPI 12 and 19, W7∆CjNC110∆metAB also demonstrated significantly reduced colonization when compared to WT (*P* < 0.05); this change was similar in magnitude to the reduced colonization seen with IA3902∆CjNC110. Critically*,* the addition of *metAB* restored colonization in IA3902∆CjNC110::metAB to WT levels by DPI 12 and 19 (*P* > 0.05). These results confirm that sRNA CjNC110 positively regulates the core conserved AMC, and L-met availability is critical for chick colonization in *C. jejuni*. In W7, the secondary MetAB system enables L-met availability to be restored to overcome the regulatory disruption to the AMC when CjNC110 is deleted, explaining the divergent chick colonization phenotype seen between W7∆CjNC110 (MetAB present) and IA3902∆CjNC110 (MetAB absent).

## DISCUSSION

In this study, the regulatory action and conservation of function of the sRNA CjNC110 between two different zoonotically important *C. jejuni* strains of distinct origin (human gastroenteritis vs sheep abortion) were examined. Our results demonstrated a clear conserved function for the regulation of several key virulence-associated phenotypes by sRNA CjNC110 in *C. jejuni.* While phenotypic differences were exhibited in chicken colonization and L-met levels between strains, we clearly demonstrated that the presence of a secondary pathway for generation of L-met via MetAB in W7 and not in IA3902 explains this difference and confirms a conserved role for regulation of the AMC by CjNC110 in *C. jejuni*. This result is similar to our previously published findings for disruption of the AMC with *luxS* deletion where the addition of MetAB was also able to rescue L-met production and chicken colonization phenotypes (31). These results also support our working hypothesis that while IA3902 and W7 share a core conserved regulatory network modulated by CjNC110, differences exist for some phenotypes due to variability in genomic content between strains.

The AMC is vital for the production of both L-met and SAM in bacteria (26, 28). In *C. jejuni*, L-met biosynthesis occurs by the conversion of L-homocysteine and methyltetrafolate (CH_3_H_4_folate) to L-met utilizing enzymes MetEF (28). L-homocysteine can be derived through two independent pathways: (i) conversion of S-ribosylhomocysteine via LuxS within the AMC (conserved), and (ii) conversion of homoserine via the MetABC pathway if all components of the MetABC system are present (not present in all strains) (31). Bacteria then utilize L-met to initiate protein translation, build proteins, and generate SAM within the AMC (26, 43). In most bacteria, regulation of the AMC has been demonstrated to occur through feedback mechanisms for several AMC metabolic by-products and/or effectors, as well as DNA-binding protein regulators such as MetR and MetJ that regulate at the transcriptional level (28). BlastN and BlastP analysis reveals, however, that *C. jejuni* lacks met-box DNA consensus sequences within promoter regions and genes encoding for protein homologs of both MetJ and MetR, as well as other known regulators such as SahR and SamR (44–46). Additionally, comparative genomic analysis of known methionine pathways in NCTC 11168 and IA3902 via the Rfam database (47) for the three families of riboswitches, which have also been shown to regulate genes for methionine metabolism (SAH, SAM-alpha, and SAM_SAH) (48–51) demonstrated that none of these riboswitches are present. In *C. jejuni*, HeuR is a DNA-binding protein that has been demonstrated to negatively regulate *metC* which is part of the auxiliary MetABC pathway, but HeuR has not been shown to regulate the core methionine biosynthesis pathway (52). Thus, the identification of sRNAs regulating within the AMC represents the only known regulators of the core conserved portion of this pathway in *C. jejuni* to date.

**Fig 6:**
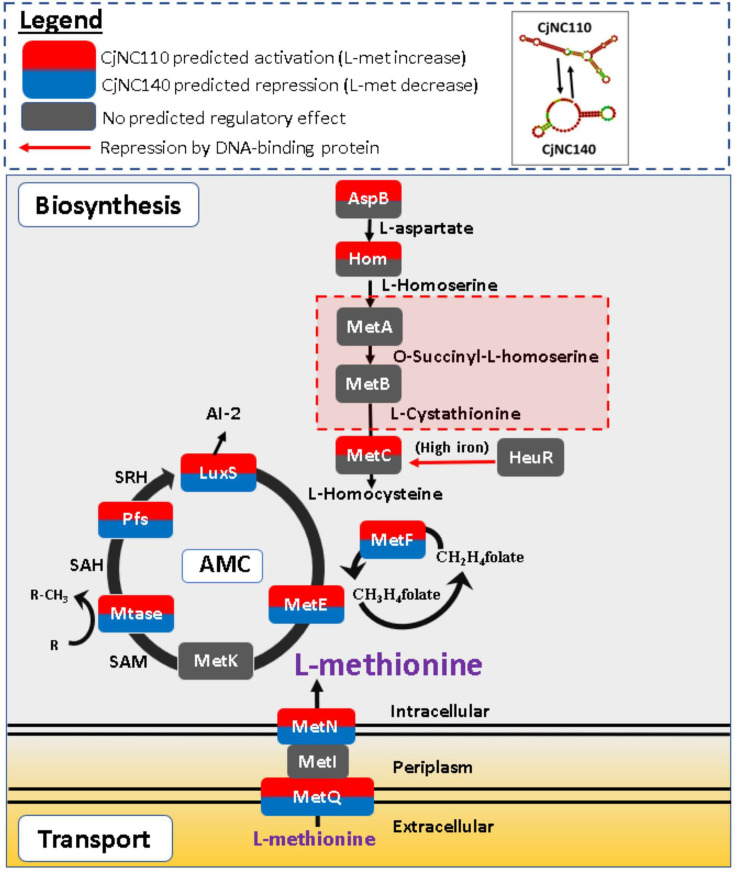
A predicted model for the role of sRNAs CjNC110 (activator) and CjNC140 (repressor) in regulation of intracellular L-met levels in *C. jejuni*. Model is based on computationally predicted binding sites with high-binding energy scores within the 5′ UTR of the mRNA to a stem-loop region of the sRNA. IA3902 does not encode *metAB* (illustrated by the red box with dashed lines) and is not expected to utilize this pathway. Each box represents an enzyme, and the primary substrate metabolite for each enzyme is provided as text. Enzyme box color indicates computationally predicted direct regulatory effect by CjNC110 (top half) or CjNC140 (bottom half): (i) basal expression is indicated by gray color fill, (ii) activation by red color fill, and (iii) repression by blue color fill.

Our data suggest that CjNC110 serves to increase intracellular L-met biosynthesis through conserved regulation of key AMC-associated genes in *C. jejuni*, including potential direct interactions with *luxS*, *metE*, *metF*, and *pfs* as binding is predicted to occur at the 5-UTRs of these mRNAs via SL1 of CjNC110. The SL1 of CjNC110 has been demonstrated to be an important interaction region for RNA-RNA binding, as CjNC110 was previously shown to bind to sRNA CjNC140 using SL1 (24). As CjNC140 has also been demonstrated to function to decrease intracellular L-met levels, and IntaRNA computational and RNAseq analyses strongly indicate that CjNC140 represses *metE*, CjNC110 may also regulate the AMC indirectly through interactions with CjNC140 (24). Thus, CjNC110 and CjNC140 may serve as a checks and balances system for methionine biosynthesis in *C. jejuni*, as the inactivation of either dysregulates the normal feedback mechanisms of the AMC that control L-met and SAM levels (see summary of predicted model in Fig. 6). Regulation of *metF* by CjNC110 may normally act to increase CH_3_H_4_folate production and subsequent downstream L-met production by MetE. Both the core AMC and the MetABC pathway require MetE activity to produce L-met; therefore, our data demonstrate that regulation of this gene alone does not explain the regulatory role of CjNC110. While our phenotypic data utilizing the MetAB system indicate that CjNC110 normally serves to activate the AMC and L-met production, our computational data suggest that several other mechanisms for altered L-met availability may also be regulated by CjNC110, including increased L-met importation and increased turnover/usage of L-met and SAM by methyltransferases. The possibility of concurrent altered regulation of L-met importation unfortunately makes further verification of our findings through exogenous L-met addition not feasible. Thus, further research is needed to directly examine the RNA:RNA interactions that lead to the observed phenotypes to more fully validate the exact mechanisms by which CjNC110 and/or CjNC140 are involved in fine-tuning both L-met biosynthesis and importation depending upon *C. jejuni* cellular requirements.

The main phenotypic difference between *C. jejuni* W7 and IA3902 CjNC110 isogenic mutants that was not investigated further was the observed difference in H_2_O_2_ sensitivity. Inactivation of CjNC110 in IA3902 was previously shown to lead to increased H_2_O_2_ sensitivity (23), while during this study, no phenotypic changes were seen in W7∆CjNC110. While this phenotypic difference could play a role when mounting an oxidative stress response within animal hosts (53), our MetAB mutagenesis strategy indicates that this was not the primary cause of the difference seen in chicken colonization ability. Further investigation of the function of oxidative stress genes and the regulation of oxidative stress genes by sRNA CjNC110 in *C. jejuni* is warranted.

Finally, the motility and autoagglutination assays strongly indicate that regulation of these critical phenotypes by CjNC110 is also conserved in *C. jejuni.* Both motility and autoagglutination have been demonstrated to be key virulence traits of *Campylobacter* enabling the colonization of the intestinal mucosal lining and biofilm formation (54, 55). In both W7 and IA3902, flagellar- and glycosylation-associated mRNAs were computationally predicted via the global search to be regulated by CjNC110. Thus, the high conservation of CjNC110, which is predicted to target flagella-associated mRNAs, strongly correlates with the *C. jejuni* phenotypic trends after the inactivation of CjNC110 and also warrants further investigation.

In summary, we demonstrated that positive regulation by CjNC110 of AMC biosynthesis contributes to L-met availability and further supports AMC turnover as a key factor driving *C. jejuni* chicken colonization. For future studies, our results indicate that the genomic reduction in IA3902 (lacking the secondary MetAB system) provides a simpler model for further investigation of AMC regulators, metabolic end-points, and gene functions in relation to *C. jejuni* pathogenesis because the core AMC of *C. jejuni* remains intact in the absence of a secondary system for L-met generation. As a growing body of evidence suggests that maintenance of L-met availability and AMC homeostasis appears to be critical for *C. jejuni* colonization, interventions targeting the AMC could provide a critical control point for therapeutic drug options to combat this zoonotic pathogen.

## MATERIALS AND METHODS

### Bacterial strains, growth conditions, plasmids, and primers

Representative *C. jejuni* SA clone IA3902 and *C. jejuni* W7, a motile variant of *C. jejuni* NCTC 11168, were utilized during this study (11, 13, 14). *C. jejuni* IA3902 WT and W7 WT, as well as their isogenic mutants, were grown in MH broth or on MH agar plates (Becton-Dickinson, Franklin Lakes, NJ) at 42°C under microaerophilic conditions as previously described (23). For strains resistant to specific antibiotic(s) due to selective markers, chloramphenicol (5 µg/mL), kanamycin (50 µg/mL), and/or apramycin (20 µg/mL; Thermo Fisher Scientific, Waltham, MA, USA) were added to MH broth or agar plates when required. All strains used are listed in Table S2. Primer sequences and the non-radioactive digoxigenin (DIG) labeled locked nucleic acid (LNA) CjNC110 probe sequence (Qiagen, Hilden, Germany) are listed in Table S3. Purified plasmids and extracted DNA utilized during this study are found in Table S4.

### Creation of *C. jejuni* W7ΔCjNC110, W7ΔCjNC110c, W7ΔluxSΔCjNC110, W7ΔCjNC110ΔmetAB, and IA3902ΔCjNC110::metAB

W7∆CjNC110 and IA3902∆CjNC110 were constructed previously (23). To construct the remaining mutants, DNA was extracted and purified using *Quick*-DNA Miniprep Kit (Zymo Research, Irvine, CA, USA) according to the manufacturer’s instructions. Following DNA extraction, natural transformation (56–58) using 5 µg of purified DNA from IA3902∆CjNC110c was used to move the CjNC110 intergenic region containing the insertion of CjNC110 into *C. jejuni* W7 to create W7∆CjNC110c (Table S4 and Fig. S6). Next, 5 µg of purified genomic DNA from the previously created insertional *luxS* mutant *C. jejuni* W7∆luxS (14) was used for homologous recombination in W7∆CjNC110 to create a double knockout mutant W7∆luxS∆CjNC110. Both the intergenic region downstream of *luxS*, where CjNC110 is located, and the coding sequence of *luxS* was confirmed to be 100% homologous between IA3902 and W7 based on BlastN analysis prior to transfer (14)*.* Lastly, electroporation (58) was used to move the previously constructed *metAB* deletion (pUC19::ΔmetAB) or *metAB* insertion (pRRC::metAB) into *C. jejuni* ΔCjNC110 backgrounds, creating W7∆CjNC110∆metAB and IA3902∆CjNC110::metAB as previously described (31) (Table S3 ). DNA from positive clones was PCR amplified using a high fidelity TaKaRa Ex Taq DNA Polymerase (TaKaRa Bio Inc, San Jose, CA, USA), PCR products purified using a QIAquick PCR Purification Kit (Qiagen, Hilden, Germany), and Sanger sequenced at the Iowa State University (ISU) DNA facility (Ames, Iowa). All PCR-positive isogenic mutants used during this study were screened for the presence of motility and compared to their respective WT controls as described below.

### Growth curves using strains IA3902, W7, and their isogenic mutants

For each growth curve, the growth over 30 h was analyzed to determine the average optical density (*A*_600_) and average CFUs per milliliter using three independent assays, collecting one sample per strain per time point for each assay, as previously described (23). During the W7 growth curve, only W7 strains were utilized, which included W7 wild-type, W7∆CjNC110, W7∆CjNC110c, W7∆luxS, and W7∆luxS∆CjNC110. Overnight cultures were normalized to a starting *A*_600_ of 0.05 using a GENESYS 10S VIS Spectrophotometer (Thermo Scientific, Waltham, MA) and incubated microaerobically at 42°C with constant shaking at 125 rpm. Next, samples were collected at pre-designated time points (0, 4, 8, 12, 14, 16, 20, 24, 28, and 30 h). A second growth curve used for collection of samples for TR-FRET assays as described below followed the same protocol but used both IA3902 WT and W7 WT, as well as the isogenic mutants, W7∆CjNC110, W7∆CjNC110c, W7∆CjNC110∆metAB, IA3902∆CjNC110, IA3902∆CjNC110c, and IA3902∆CjNC110::metAB.

The CFUs of each biological group, corresponding to the collection time points stated above for each growth curve, were also determined via the drop-plate method (59). Two-way ANOVA with repeated measures, in conjunction with Tukey’s multiple-comparison testing, was used to analyze *A*_600_ over time. For both growth curves, independent two-way ANOVA with repeated measures using the average CFU per milliliter (log_10_) over time, which demonstrated no statistical (*P* > 0.05) difference in growth between strains. The statistical tests utilized required a *P*-value of <0.05 to reach significance. Culture processing of RNA for the northern blot, as well as quantification of L-met and SAM metabolites, are described below.

### RNA extraction and northern blotting

Total RNA was extracted using QIAzol lysis reagent (Qiagen, Hilden, Germany) from cell cultures corresponding to the W7 growth curve, specifically using samples from W7 WT, W7ΔNC110c, and W7ΔNC110 at early stationary phase of growth (14 h), as previously described (23). For northern blot detection, total RNA from bacterial cells (15 µg) and a pre-stained DynaMarker RNA High Ladder (Diagnocine LLC, Hackensack, NJ, USA) were loaded onto 1.3% denaturing agarose (Bio-Rad, Hercules, CA, USA) and electrophoresed, followed by RNA transfer to a positively charged nylon membrane (Roche, Basel, Switzerland). Next, 25 ng/mL of LNA-DIG probe CjNC110 (/5’DigN/GCACATCAGTTTCAT/3’Dig_N/; Qiagen, Hilden, Germany) was added to DIG EasyHyb Buffer (Roche, Basel, Switzerland), and hybridization took place followed by several washing steps. For the detection of sRNA transcript CjNC110, the ChemiDoc Imager was used (Bio-Rad, Hercules, CA, USA). ImageLab software (v3.0.1, Bio-Rad, Hercules, CA, USA) was utilized to generate a standard curve using the relative front and nt size of the ladder to predict the average band size of the target RNA as previously described (23). The original northern blot detection image is provided in Fig. S7.

### Enumeration of L-met and SAM concentrations

Samples from the second growth curve, including W7 WT, IA3902 WT, W7∆CjNC110, W7∆CjNC110c, W7∆CjNC110∆metAB, IA3902∆CjNC110, IA3902∆CjNC110c, and IA3902∆CjNC110::metAB, were collected at time points 12 h (IA3902) and 14 h (W7) and used for L-met and SAM metabolite quantification. Preliminary testing revealed the *A*_600_ for the W7 strains to be lower during log phase and early stationary phase compared to IA3902 strains. Hence, sample collection was staggered to ensure similar CFU per milliliter counts at the early stationary phase (12 h, IA3902; 14 h, W7) as previously described (31). L-met and SAM metabolite quantification was conducted using TR-FRET kits: Bridge-It L-methionine Fluorescence Assay Kit and Bridge-It S-adenosylmethionine Fluorescence Assay Kit (Mediomics, St. Louis, MO, USA), as previously reported by our laboratory (31). Samples were run in triplicate from each growth curve, and fluorescence was measured using the FLUOstar Omega Microplate Reader (BMG Labtech, Ortenburg, Germany). L-met concentrations were calculated using a standard curve utilizing internal L-met serial dilution controls (31). Both L-met and SAM average values were evaluated via one-way ANOVA with Tukey’s multiple-comparison testing; a *P*-value threshold of <0.05 was required to reach significance.

### Motility, autoagglutination, and hydrogen peroxide sensitivity phenotype assays

The motility, autoagglutination, and hydrogen peroxide sensitivity assays were performed as previously described (23, 24) using W7 strains, which included W7 WT, W7∆CjNC110, W7∆CjNC110c, W7∆luxS, and W7∆CjNC110∆luxS. All assays were performed using three independent studies with at least three technical replicates to obtain average values per strain. All *A*_600_ culture normalizations were conducted using a GENESYS 10S VIS Spectrophotometer (Thermo Scientific, Waltham, MA). These phenotype assays were statistically analyzed using one-way ANOVA or two-way ANOVA followed by Tukey’s multiple-comparison testing as appropriate; a *P*-value threshold of <0.05 was required to reach significance.

### *C. jejuni* chicken colonization studies

All studies involving animals were approved by the ISU Institutional Animal Care and Use Committee (IACUC protocols 19-256 and 22-173) and followed all appropriate animal care guidelines. Each chicken colonization study was performed as previously described (23, 24). For the first chicken colonization study, W7 WT, W7∆CjNC110, W7∆CjNC110c, W7∆luxS, and W7∆CjNC110∆luxS were utilized. During the second chicken colonization study, W7 WT, IA3902 WT, W7∆CjNC110, W7∆CjNC110∆metAB, IA3902∆CjNC110, and IA3902∆CjNC110::metAB were used. For both chicken colonization studies, 3-day-old commercial broiler chickens were randomly assigned to treatment groups consisting of a minimum of 18 birds per group. Chicks were confirmed to be *Campylobacter*-free via screening of cloacal swabs by culture. Subsequently, chickens were orally inoculated with 200 µL (1 × 10^7^ CFU) of bacterial suspension via oral gavage (Fig. S3). Colonization levels were determined at DPI 5, 12, and 19. Cecal contents were collected per bird at each DPI, and then (wt/vol) normalized for 10-fold serial dilutions before plating on MH agar supplemented with *Campylobacter* Selective Supplement (SR0204E; Oxoid, Thermo Fisher Scientific, Waltham, MA, USA) and Growth Supplement (SR0232E) (Oxoid, Thermo Fisher Scientific, Waltham, MA, USA). Following serial dilutions of cecal contents, the target CFU range was set at 30–300 per bird for accurate quantification of CFU per gram. Next, CFU per gram was log_10_ transformed, and the geometric mean for each strain was taken at each DPI.

For statistical analysis, independent one-way ANOVA tests at each DPI were performed in conjunction with Tukey’s multiple-comparison tests to determine if there were significant differences (*P* value < 0.05) in colonization ability between strains. A Shapiro-Wilk test was performed for each DPI to confirm normal distribution and adequate sample size (*N*) representation per group (*P* values > 0.05). To test for equality of group variances, based on performing ANOVA, a Brown-Forsythe test was performed at each DPI; the equal variances assumption was confirmed to be valid at each DPI (*P* values > 0.05). Inoculum suspension was confirmed to be an average of ~1 × 10^7^ CFU/mL for each respective strain, and statistical differences (*P* > 0.05) in inoculum were not detected using one-way ANOVA analysis matching trends described previously (23).

### BlastN, BlastP, IntaRNA, and RNAup analyses

The Basic Local Alignment Search Tools BlastN and BlastP (https://blast.ncbi.nlm.nih.gov/) were used against the genomes or proteomes of both *C. jejuni* IA3902 (NC_017279.1) and *C. jejuni* W7 (reference genome NCTC 11168; NC_002163). BlastN was used to obtain non-coding sequences of sRNA CjNC110 (137b) and for select extraction of genome regions with genes of interest (15). Freely available IntaRNA (http://rna.informatik.uni-freiburg.de/IntaRNA/Input.jsp) was used to determine the top predicted regulatory partner mRNA interactions with CjNC110 (137b and 226b transcripts) (35). The top mRNA partners with the lowest free energy of binding (kcal/mol) were further analyzed as previously described (24). Briefly, the 5′- and 3′-UTR from −75 to +75 nt from the start or stop codon, respectively, were analyzed to obtain a top predicted mRNA partner list for CjNC110. The overlapping core global regulatory network of CjNC110 was then combined for both *C. jejuni* IA3902 and *C. jejuni* W7 aligned by homologous locus tags between strains. For computational analysis of select mRNA partners using IntaRNA (35) or RNAup (36), the same settings were used −75 to +75 nt from the start or stop codon to examine potential binding at the 5′-UTR using single target input mode. Protein structural information was searched for within UniProt (reference proteome *C. jejuni* 11168, UP000000799). GraphPad (GraphPad Prism) was used for statistical testing as described above and to create graphical annotations using the layout function.

## Data Availability

All phenotype data generated, as well as all strains and primers used during this study, are provided in File S1.

## SUPPLEMENTAL MATERIAL

Supplemental material msphere.00832-24-s0001.pdf Supplemental tables and figures.

ASM does not own the copyrights to Supplemental Material that may be linked to, or accessed through, an article. The authors have granted ASM a non-exclusive, world-wide license to publish the Supplemental Material files. Please contact the corresponding author directly for reuse.
