# Supplementary material for: Small RNA CjNC110 regulates the activated methyl cycle to enable optimal chicken colonization by Campylobacter jejuni
Source: mSphere. 2025 Jan 8;10(1):e00832-24. doi: 10.1128/msphere.00832-24 (PMC11774046; doi:10.1128/msphere.00832-24)
Supplement: Supplemental material — Supplemental tables and figures. [file msphere.00832-24-s0001.pdf]

SUPPLEMENTAL FILE 1

Supplemental Tables

Table S1. IntaRNA globally predicted conserved targeting of flagella, H_2O_2, and activated methyl cycle-associated mRNAs in *C. jejuni*

		Binding Energy (kcal/mol)[a]				Binding Energy (kcal/mol)[a]	
**mRNA**	**UTR**	**IA3902**	**W7**	**mRNA**	**UTR**	**IA3902**	**W7**
**Motility**				**H_2O_2 Stress Response**			
flgS	5'	-21.6	-21.6	*sodB*	5’	-7.59	-7.59
flaG	5'	-12.1	-12.1	*perR*	5’	-6.12	-6.12
flgK	5'	-11.3	-11.3				
cheQ	3'	-10.4	-10.4				
fliI	3'	-7.5	-7.4				
cetB	3'	-6.4	-6.4				
fliI	5'	-5.5	-5.5				
fliN	5'	-4.9	-4.9				
**Glycosylation**				**Activated Methyl Cycle**			
waaV	3'	-14.0	-14.0	*methyltransferase*	5’	-10.36	-5.28
pglE	5'	-11.7	-11.7	*hom*	5’	-6.42	-6.42
pglJ	3'	-11.5	-11.5	*luxS*	5’	-6.07	-6.07
pglB	5'	-10.5	-10.5	*metF*	5’	-6.27	-6.27
pflB	5'	-10.2	-10.2	*metQ4*	5’	-5.06	-5.04
pglA	3'	-7.3	-7.4	*aspB*	5’	-4.96	-4.96
pglC	5'	-7.3	-7.2				
cgpA	5'	-6.9	-6.9				
wlaN	3'	-6.4	-6.4				
pglK	5'	-6.2	-6.2				
ptmA	5'	-5.4	-5.4				

[a] Free energy required to unfold the interaction site for accessibility (deltaG or kcal/mol); a lower k/cal/mol is more favorable. mRNAs and binding energy scores were in the top 325 CjNC110 partner list determined by IntaRNA global search only.

Table S2. Bacteria strains used in this study

Strains	Description	Reference
Campylobacter jejuni		
W7	Wild type motile variant of NCTC 11168	(1)
W7ΔluxS	ΔluxS::Kan^R	(1)
W7ΔCjNC110	ΔCjNC110::Cm^R	(2)
W7ΔCjNC110ΔluxS	ΔCjNC110::Cm^R ; ΔluxS::Kan^R	This Study
W7ΔCjNC110c	ΔCjNC110::Cm^R; CjNC110::Kan^R	This Study
W7ΔCjNC110ΔmetAB	ΔCjNC110::Cm^R; ΔmetAB::Apr^R	This Study
IA3902	Wild type *C. jejuni* sheep abortion (SA) clone	(3)
IA3902ΔCjNC110	ΔCjNC110::Cm^R	(2)
IA3902ΔCjNC110c	ΔCjNC110::Cm^R; CjNC110::Kan^R	(2)
IA3902ΔCjNC110::metAB	ΔCjNC110::Cm^R; ::metAB::Apr^R::Cm^R	This Study
Escherichia coli		
DH5α	*fhuA2* Δ(*argF-lacZ*)U169 *phoA gln*V44 Φ80 Δ(*lacZ*)M15 *gyrA*96 *recA1 relA1 endA1 thi-1 hsdR*17	NEB

Kan^R = Kanamycin resistance cassette

Cm^R = Chloramphenicol resistance cassette

Apr^R= Apramycin resistance cassette

Table S3. Primers used

Primers[a]	Sequence 5'-3'	Target	Use	Reference
Cj1198F1	GTTGGATCCCCATTATTAGACAGCTTTAAAGT	*luxS*	PCR	(5)
Cj1198R3	TAATCTGCAGTTTAAGCATTCTCGAGTTTTT	*luxS*	PCR	(5)
CjNC110F2	TTTGATTTGCGTTTTTGCAT	CjNC110	PCR	(3)
CjNC110R2	ATCAAGAGCTTGAGCGAAGG	CjNC110	PCR	(3)
Cj1198F1	AACTACTTCAAACATAAAATTTCCTTG	*luxS*/CjNC110	PCR	(3)
Cj1199R3	CCATGCAAAACCGGTAAAAAA	*luxS*/CjNC110	PCR	(3)
CjNC110c1F	GCAATCTAGATGCATTCTTTAGATGAAGCCA	CjNC110c	PCR	(3)
CjNC110c1R	GACTGTCTAGAAATTCTTTGCCAAGTTTGAA	CjNC110c	PCR	(3)
pRRKconF1	ATCGTAGATCAGCCATGCTA	16s/23s	PCR	(4)
METABCONR1	CAGGACTTGCTTCAGGTGTT	16s/23s	PCR	(4)
METABCONF1	CAGGACTTGCTTCAGGTGTT	*metAB*	PCR	(4)
METABCONR1	TGCTTGGCACGCTTAATCTA	*metAB*	PCR	(4)
CjNC110-LNA	/DigN/ GCACATCAGTTTCAT/Dig_N/	CjNC110	Northern	(3)

[a] pRRKconF1 and METABCONF1 used as sequencing primers.

Table S4. DNA sources for genetic manipulation

DNA/plasmid	Description	Source
IA3902ΔCjNC110	[a]DNA to transform W7 wild-type and W7ΔluxS	(3)
IA3902ΔCjNC110c	[a]DNA to transform W7ΔCjNC110 for complementation	(3)
pUC19::Δ*metAB*::Apr^r	[b]Plasmid pUC19 carrying *metAB* deletion via insertional deletion using Apr^r	(4)
pRRC::*metAB*::Apr^r	[b]Plasmid pRRC carrying *metAB* insertion with Apr^r selective marker	(4)

[a] DNA for natural transformation
[b] Plasmids for electroporation

Supplemental Figures

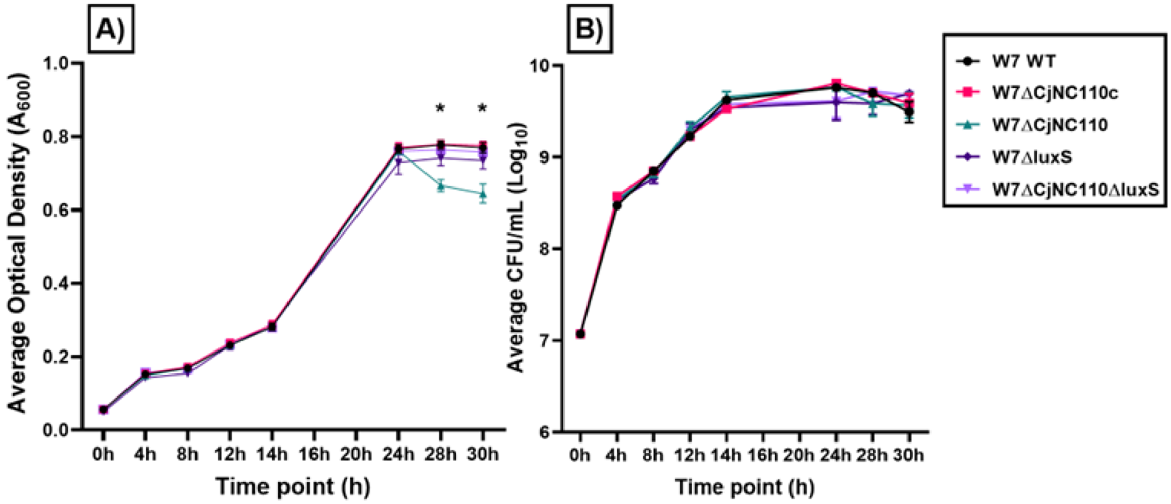

FIG S1. Growth curves reveal growth in MH media is comparable between W7 wild-type (WT) and isogenic mutants (values indicate mean ± SEM at each point). **A)** Displayed are the average optical density (A_{600}) readings over time for each strain, one measurement per timepoint was collected from three independent studies. Each shape with a corresponding color indicates the strain utilized (labeled in black box on the right). Growth curves were performed in standard MH broth using 250 mL Erlenmeyer flask shaking at 125 RPM. Analysis via two-way analysis of variance (ANOVA) of average A_{600} over time demonstrated no significant difference in growth between strains (P>0.05), for all time points except at 28 h and 30 h when comparing W7 WT to ΔCjNC110. **B)** The average colony forming units per mL (CFUs/ml) after $\log_{10}$ transformation were determined for each strain using the drop plate method with three technical replicates for each growth curve (6). No significance (P>0.05) was demonstrated when comparing each strain over time using one-way ANOVA. For each analysis, significance (P<0.05) is denoted by "*".

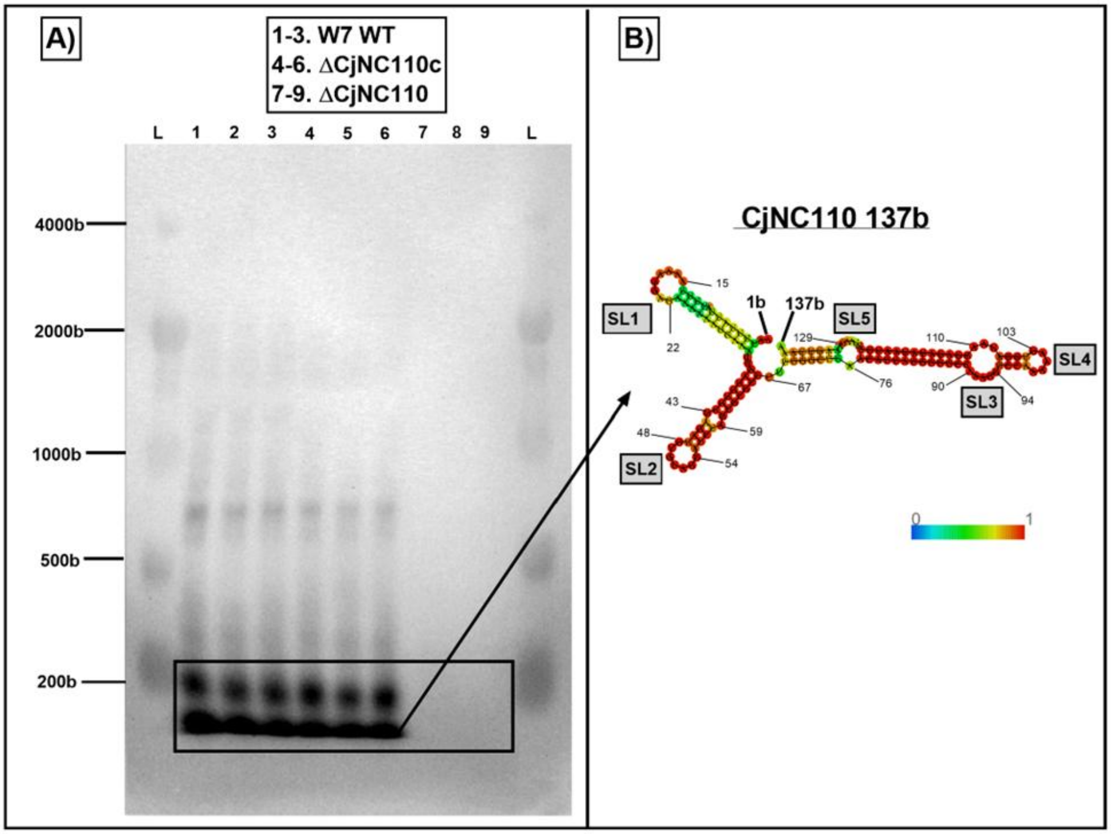

FIG S2. Northern blot reveals small RNA CjNC110 is transcribed by W7 wild-type (WT). (A) All strains used are indicated and corresponding replicates are indicated at the top of the image. L, pre-stained RNA ladder (4000-200b). Cultures for RNA extraction were collected at early-stationary phase of growth (14 h) from three separate experiments per strain tested. Northern blotting was performed using 15 μg of total RNA. The arrow indicates the dominant band detected, which corresponds to CjNC110 (137b), as demonstrated previously in *C. jejuni* (3, 7). **B)** The 137b CjNC110 RNA sequence was input within RNAfold to reveal the secondary structure of CjNC110, using minimum free energy and partition function. The base-pair probability of sRNA secondary structure formation is colored (red=high; blue=low). The RNA bases (b) from 5'-3' are indicated with black lines (-#). Grey Colored boxes indicate stem-loops (SLs). GraphPad Prism was used to annotate the predicted SLs.

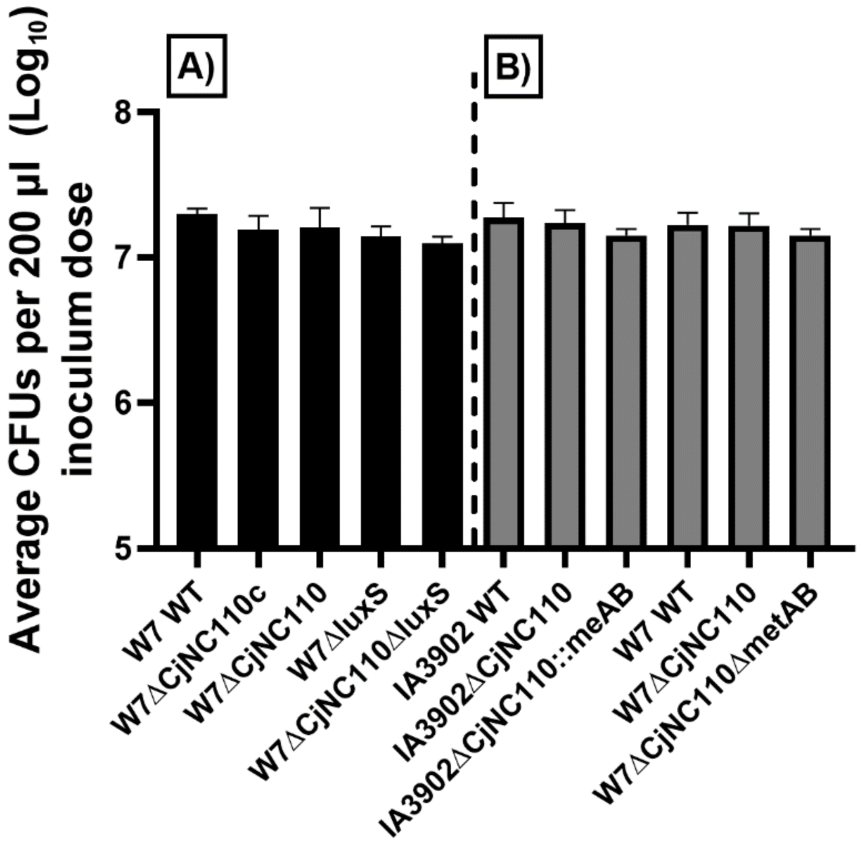

FIG S3. The initial chicken inoculum dose was similar for each strain utilized, for both the W7 (**A**, black; FIG. 1) and *metAB* (**B**, grey; FIG. 5) chicken colonization study (mean ± SEM). For both A and B, the drop-plate method was used to determine CFUs using the average of three technical replicates, after $\log_{10}$ transformation of the CFUs (6). Independent statistical analysis was conducted for each chicken colonization study. For both A and B, statistical analysis via one-way ANOVA testing demonstrated no significant difference between the strains (P>0.05). The initial inoculum dosage per strain for each independent chicken study was similar ~10^7.

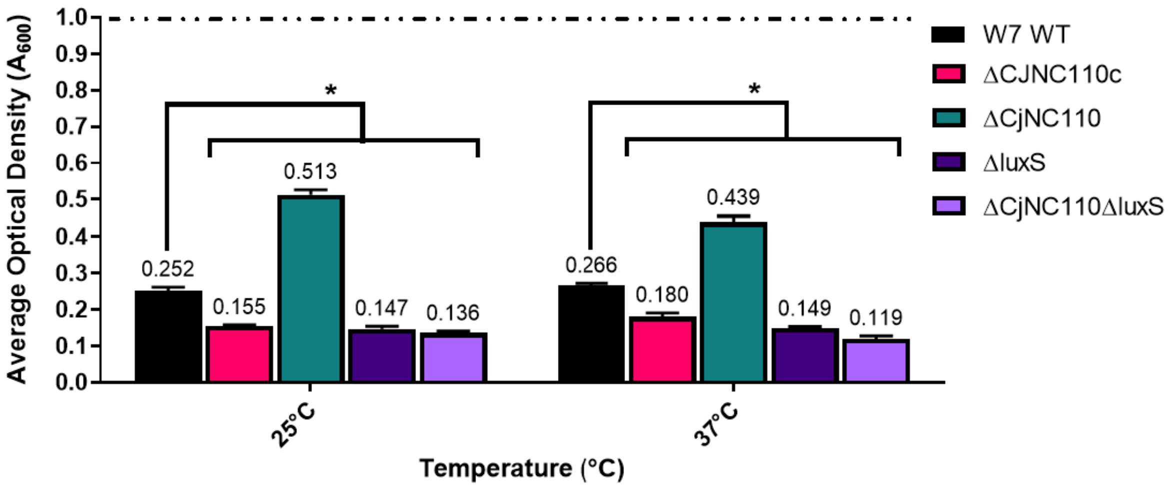

FIG S4. ΔCjNC110 decreases autoagglutination, while ΔluxS bolsters autoagglutination relative to W7 wild-type (WT) [mean ± SEM]. Colored bars indicate the average motility or autoagglutination of each strain tested at 24 hours, using a minimum of at least three technical replicates from three independent studies. Autoagglutination activity was determined by optical density (A_{600}) at 25ºC and 37ºC. All cultures started at an A_{600} of 1.0, indicated by the black dashed line. An increase in A_{600} correlates to decreased autoagglutination ability, and a decrease in A_{600} correlates to increased autoagglutination ability. For statistical analysis, two-way ANOVA with Tukey's multiple comparison test was performed. Significance ($P < 0.05$) is denoted by "*" when comparing respective strains (black lines) at each independent temperature utilized.

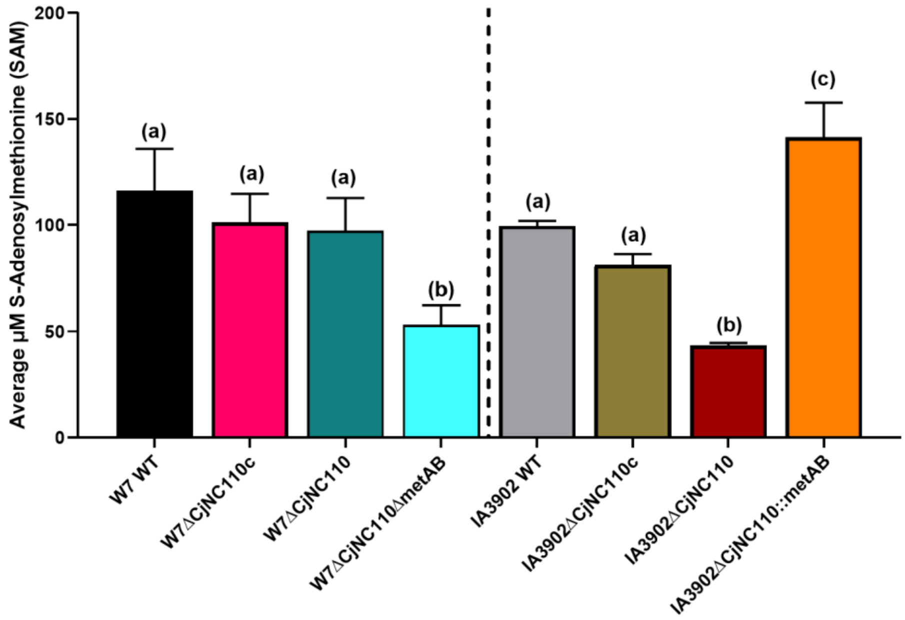

FIG S5. IA3902ΔCjNC110::metAB restores SAM production in IA3902ΔCjNC110, while IA3902ΔCjNC110ΔmetAB reduces SAM concentration to comparable levels observed for IA3902ΔCjNC110 *in vitro* (mean ± SEM). Each bar represents the average SAM metabolite concentration of each strain tested using three technical replicates from three independent studies. Strains utilized are indicated at the bottom (x-axis). L-met concentrations were calculated using a standard curve of L-met serial dilution controls. For statistical analysis, one-way ANOVA with Tukey's multiple comparison test was performed. Significance ($P < 0.05$) is denoted by "*" when comparing respective strains (dashed line). The dashed line was utilized to separate by strain either W7 or IA3902. An overlap in letters above each bar indicates no significance ($P > 0.05$) detected. All cultures were grown and collected from the same growth curves.

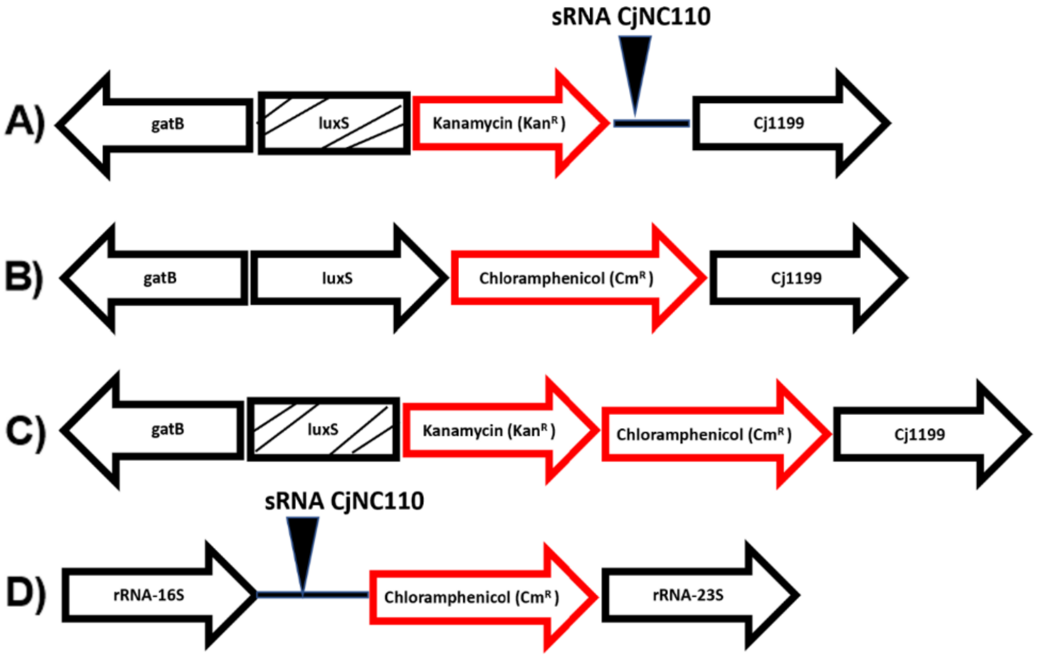

FIG S6. Illustration of isogenic mutant constructs of *C. jejuni* W7, corresponding to the genomes depicted in A-D. **A)** W7ΔluxS, *luxS* insertional deletion with small RNA (sRNA) CjNC110 present **B)** W7ΔCjNC110, Small RNA CjNC110 deletional knockout **C)** W7ΔluxSΔCjNC110, *luxS* insertional deletion and small RNA deletional knockout **D)** W7ΔCjNC110c, small RNA CjNC110 insertion construct to complement ΔCjNC110. Red colored arrows indicate genomic insertions via homologous recombination. Boxes with strike-through indicate gene-specific insertional deletions. Blimp black arrows indicate the location of sRNA CjNC110, which is located in the genome of W7 wild-type. The genomic regions depicted are 100% homologous in wild-type IA3902 and W7. PCR amplification and Sanger sequencing validated each mutant construct.

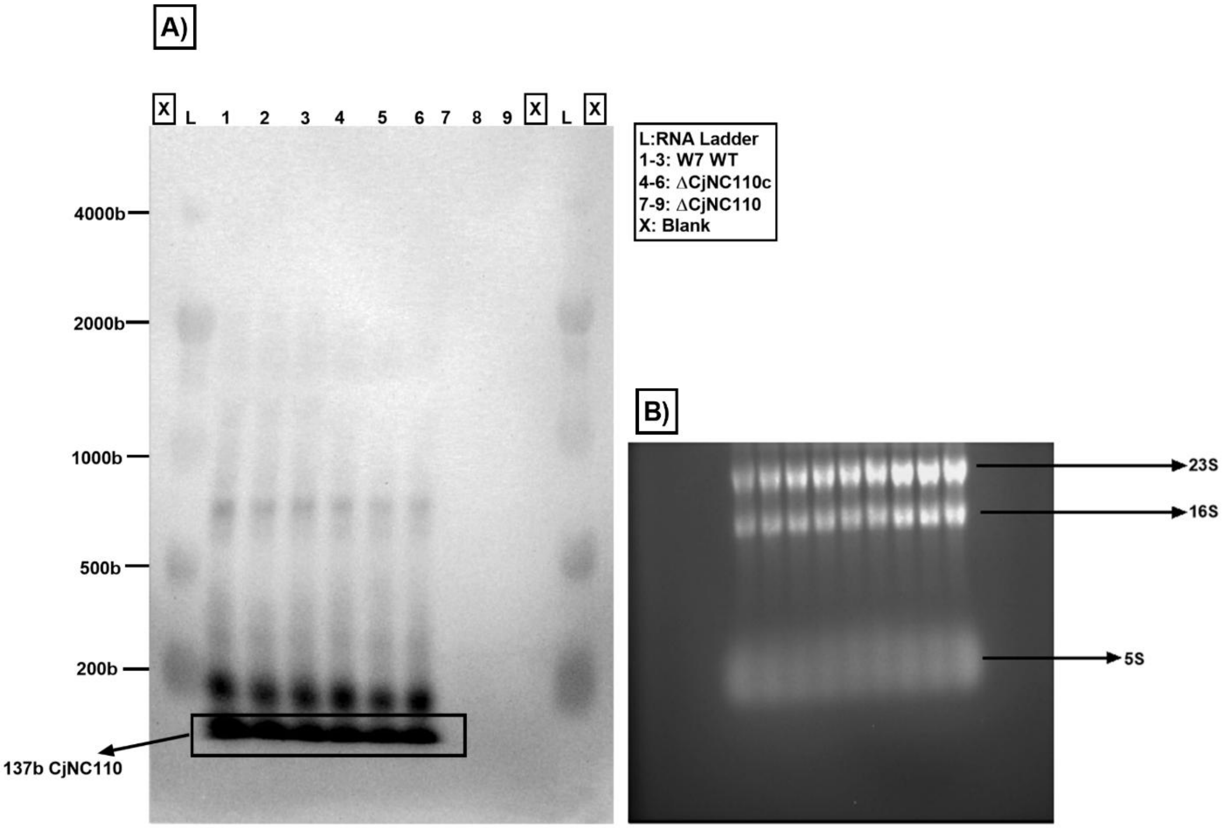

FIG S7. Original northern blot detection image and corresponding RNA quality control. **A)** Northern blot demonstrates CjNC110 is transcribed by W7 and W7ΔCjNC110c but is no longer transcribed in W7ΔCjNC110. Lane numbers indicated above demonstrate the loading order. RNA was extracted from three independent growth curves for each strain. The prominent CjNC110 band is indicated by an arrow and black box (137b). The Northern blot analysis was conducted using 15 µg of total RNA, corresponding to the RNA bands in **B)** detected by ethidium bromide staining. The CjNC110 137b band matches previously reported results (3, 7).

Supplemental References

1. Plummer P, Sahin O, Burrough E, Sippy R, Mou K, Rabenold J, Yaeger M, Zhang Q. 2012. Critical role of LuxS in the virulence of *Campylobacter jejuni* in a guinea pig model of abortion. *Infect. Immun.* 80:585–593.
2. Kreuder AJ, Ruddell B, Mou K, *et al*. 2020. Small noncoding RNA CjNC110 influences motility, autoagglutination, AI-2 localization, hydrogen peroxide sensitivity, and chicken colonization in *Campylobacter jejuni*. *Infect. Immun.* 88:1.
3. Sahin O, Plummer PJ, Jordan DM, Sulaj K, Pereira S, Robbe-Austerman S, Wang L, Yaeger MJ, Hoffman LJ, Zhang Q. 2008. Emergence of a tetracycline-resistant *Campylobacter jejuni* clone associated with outbreaks of ovine abortion in the United States. *J. Clin. Microbiol.* 46:1663–1671.
4. Ruddell B, Hassall A, Sahin O, Zhang Q, Plummer PJ, Kreuder AJ. 2020. Role of metAB in methionine metabolism and optimal chicken colonization in *Campylobacter jejuni*. *Infect. Immun.* 89.
5. Plummer P, Zhu J, Akiba M, Pei D, Zhang Q. 2011. Identification of a key amino acid of LuxS involved in AI-2 production in *Campylobacter jejuni*. *Plos One* 6:e15876.
6. Chen Y, Nace W, Irwin L. 2003. A 6×6 drop plate method for simultaneous colony counting and MPN enumeration of *Campylobacter jejuni*, *Listeria monocytogenes*, and *Escherichia coli*. *J. Microbiol. Meth.* 55:475–479.
7. Dugar G, Herbig A, Forstner KU, Heidrich N, Reinhardt R, Nieselt K, Sharma CM. 2013. High-resolution transcriptome maps reveal strain-specific regulatory features of multiple *Campylobacter jejuni* isolates. *PLoS Genet* 9:e1003495.
